# An Overview of Infectious and Non-Infectious Causes of Pregnancy Losses in Equine

**DOI:** 10.3390/ani14131961

**Published:** 2024-07-02

**Authors:** Liangliang Li, Shuwen Li, Haoran Ma, Muhammad Faheem Akhtar, Ying Tan, Tongtong Wang, Wenhua Liu, Adnan Khan, Muhammad Zahoor Khan, Changfa Wang

**Affiliations:** 1 Liaocheng Research Institute of Donkey High-Efficiency Breeding and Ecological Feeding, Liaocheng University, Liaocheng 252059, China; liliangliang@lcu.edu.cn (L.L.);; 2 College of Veterinary Medicine, Qingdao Agricultural University, Qingdao 266109, China; 3 Genome Analysis Laboratory of the Ministry of Agriculture, Agricultural Genomics Institute at Shenzhen, Chinese Academy of Agricultural Sciences, Shenzhen 511464, China

**Keywords:** equine, pregnancy loss, infectious and non-infectious factors, preventive measures

## Abstract

**Simple Summary:**

Pregnancy loss is a major economic concern for the equine industry. It can be caused by both infectious agents (equine herpesvirus, bacteria, fungi, and parasites) and non-infectious factors (like twin pregnancies, endocrine disorders, and umbilical cord torsion). The increasing number of large-scale donkey breeding operations in China has brought attention to these reproductive challenges. To prevent pregnancy loss and maintain the overall health and productivity of horses, early detection, proper management, and control measures are crucial.

**Abstract:**

Equine breeding plays an essential role in the local economic development of many countries, and it has experienced rapid growth in China in recent years. However, the equine industry, particularly large-scale donkey farms, faces a significant challenge with pregnancy losses. Unfortunately, there is a lack of systematic research on abortion during equine breeding. Several causes, both infectious and non-infectious, of pregnancy losses have been documented in equines. The infectious causes are viruses, bacteria, parasites, and fungi. Non-infectious causes may include long transportation, ingestion of mycotoxins, hormonal disturbances, twinning, placentitis, umbilical length and torsion, etc. In current review, we discuss the transmission routes, diagnostic methods, and control measures for these infectious agents. Early detection of the cause and appropriate management are crucial in preventing pregnancy loss in equine practice. This review aims to provide a comprehensive understanding of the potential causes of abortion in equines, including infectious agents and non-infectious factors. It emphasizes the importance of continued research and effective control measures to address this significant challenge in the equine industry.

## 1. Introduction

Reproductive failure is a major cause of economic losses in the equine industry. Equine abortion, which refers to the loss of pregnancy before 300 days’ gestation, presents a significant challenge for equine breeding enterprises [1,2,3,4,5]. In recent years, there has been a growing interest in equine practices, particularly large-scale donkey breeding, making the donkey industry a crucial part of husbandry in China [6,7]. Donkeys have recently emerged in China’s livestock industry due to their multiple roles, including ejiao (a traditional Chinese remedy that is made by extracting collagen from donkey skin) production [8,9,10], meat production [11,12,13], and milk production [14,15]. Despite their significance in the livestock industry, donkeys worldwide, including in China, face a range of issues, including reproductive problems [16,17].

Abortion in pregnant donkeys and mares can be caused by infectious [18,19,20,21,22,23,24,25] or non-infectious agents [26]. The infectious agents responsible for abortion in donkeys and mares include viruses (equine arteritis virus, equine herpesvirus, equine infectious anemia virus, and West Nile virus) [27,28,29], bacteria (i.e., *Salmonella*, *Streptococcus zooepidemicus*, *Escherichia coli*, *Rhodococcus equi*, *Leptospirosis*, *Chlamydia psittaci*, and Equine monocytic ehrlichiosis) [30,31,32,33,34], fungi (i.e., *Aspergillus* spp. and *Mucocuraceus* fungi) [35,36,37], and parasites (i.e., *Neosporosis* and equine piroplasmosis) [38].

Non-infectious factors include endocrine disorders, umbilical cord torsion, twinning, toxicosis, and extensive management practices. Thus, early detection of the cause of abortion and appropriate management to prevent pregnancy loss are essential in equine practice [39,40,41,42]. This paper presents an overview of the potential causes of abortion in donkeys. These causes encompass a broad range of factors, including infectious and non-infectious agents. Additionally, we discuss the diagnosis and control measures for pathogenic agents. The study contributes to the development of a theoretical foundation for preventing abortion in the donkey industry.

## 2. Infectious Risk Factors Associated with Abortion and Pregnancy Losses in Equines 

A range of infectious pathogens are responsible for abortion and pregnancy losses in equines. An overview of these infectious agents reported in previous research is provided below.

### 2.1. Viral Agents Associated with Abortion in Equines

Viral pathogens have become important factors in the study of equine abortion [43,44]. Among them, equine arteritis virus [45,46], equine herpesvirus [47,48,49], and equine infectious anemia virus [50,51] are the main viruses associated with equine abortion which lead to pregnancy loss. These studies highlight the critical impact of viral agents on reproductive health in equines, warranting comprehensive attention and research.

#### 2.1.1. Equine Arteritis Virus (EAV)

EAV is a single-stranded, positive-sense RNA virus that belongs to the family *Arteriviridae* (genus Alpharterivirus, order Nidovirales) [52]. It is an enveloped virus that specifically infects Equidae animals, including horses, donkeys, and zebras [52,53,54,55]. Infection of mares with EAV can lead to abortion, fetal death, and severe respiratory diseases [55,56]. EAV has two subtypes: North American (NA) and European (EU) [29,57]. The main mode of transmission for EAV is through respiratory secretions or close contact with infected animals. Male donkeys or stallions typically carry the virus and can transmit it to susceptible animals through breeding [58,59,60,61]. It is worth noting that a new strain of EAV, which differs from the EAV viruses found in horses and donkeys in North America and Europe, was isolated from the semen of a naturally infected South African donkey [58,62]. Additionally, a novel EAV strain was isolated from feral donkeys in Chile [29], highlighting the global threat of EAV to the donkey industry. Diagnosis of EAV involves clinical virus isolation, reverse transcription–polymerase chain reaction (RT-PCR), immunohistochemistry, and serologic tests [52,63,64,65].

#### 2.1.2. Equine Herpesvirus

Equine herpesvirus poses a significant threat to the equine industry due to its association with abortion, respiratory disease, neurologic disease, and neonatal death [66,67,68,69]. There are a total of nine identified herpesviruses (EHV-1-9). Horses are the natural hosts for EHV-1 to EHV-5, while donkeys serve as the natural hosts for EHV-6 to EHV-8, which are also known as asinine herpesviruses (AHV, AHV1-3). EHV-9 has been found in Thomson’s gazelle, giraffe, and polar bear. The Herpesviridae family is categorized into three subfamilies based on morphology and biological properties: *Alphaherpesvirinae*, *Betaherpesvirinae*, and *Gamaherpesvirinae.* Equine herpesviruses (EHVs) such as EHV-1, EHV-3, EHV-4, EHV-6, EHV-8, and EHV-9 belong to the *Alphaherpesvirinae* subfamily, while EHV-2, EHV-5, and EHV-7 are part of the *Gamaherpesvirinae* subfamily. EHV-1, EHV-7, and EHV-8 are particularly pathogenic EHVs closely associated with abortion in pregnant donkeys [66,67,68,69]. Consistently, Ali et al. [1] reported a case of EHV-1-induced abortion and neonatal deaths in pregnant mares and female donkeys in Egypt. Additionally, LeCuyer et al. [70] reported a case of abortion caused by EHV-7 in a female Mediterranean miniature donkey at the Washington Animal Disease Diagnostic Laboratory. Wang et al. [19] also documented a case of EHV-8-induced abortion in a female donkey on the 296th day of pregnancy at a large-scale farm in China. EHVs can be transmitted through aerosols, feces, and water [71]. Diagnosis of EHVs-related diseases requires a combination of clinical history, presentation, PCR, virus isolation, immunohistochemistry, and serologic tests [72,73].

#### 2.1.3. Equine Infectious Anemia Virus (EIAV)

Equine infectious anemia virus (EIAV) is responsible for causing equine infectious anemia (EIA), which leads to symptoms such as fever, viremia, thrombocytopenia, weight loss, and abortion in horses. This virus belongs to the *Retroviridae* family, specifically the Lentivirus genus. Its impact on the global horse industry is significant, resulting in substantial economic losses [74]. Although the understanding of natural EIAV infection in donkeys is limited, a recent study by Costa VMD et al. detected EIAV-infected donkeys in Brazil using agar gel immunodiffusion (AGID), ELISA, and PCR. These findings provide direct evidence of donkeys carrying EIAV [27,51,75]. EIAV is transmitted through insect vectors and via the transplacental route. Accurate diagnosis and elimination methods are crucial for effectively controlling EIAV.

#### 2.1.4. West Nile virus (WNV)

West Nile virus (WNV) is an enveloped RNA virus that belongs to the *Flaviviridae* genus. It is known to cause outbreaks of abortion and encephalitis in mares [76,77]. Currently, nine lineages of WNV have been identified, showing biological diversity [78]. Lineage 1 WNV is prevalent worldwide, including Europe, Africa, the Middle East, Australia, and America [79,80,81]. Previous studies have found WNV in donkeys in various countries, such as Turkey, Pakistan, Bulgaria, Nigeria, Algeria, Egypt, Senegal, Israel, and Palestine [79,82,83]. The transmission of West Nile virus (WNV) occurs through both horizontal routes, primarily through the bites of infected mosquitoes, and vertical routes, including intrauterine transmission from an infected mother to the developing fetus and lactation transmission from an infected mother to her nursing infant [84,85,86,87]. The general diagnostic criteria for WNV infection in equines include PCR and ELISAs [88,89]. The methods for controlling WNV depend on vaccination in horses and vector control [84,90,91].

### 2.2. Bacterial Agents Associated with Equine Abortion

Besides viral infections, bacterial pathogens are also responsible for a significant number of abortions in horses and donkeys. Based on available published data, *Salmonella*, *Streptococcus*, *Escherichia*, *Rhodococcus equi*, and *Leptospira* are considered the main bacterial causes of equine abortion.

#### 2.2.1. *Salmonella*

*Salmonella* is a significant zoonotic disease worldwide, affecting a wide range of hosts, including humans, reptiles, farm animals, and insects [92,93]. It belongs to the Enterobacteriaceae family and can cause abortion, polyarthritis, and neonatal sepsis in sheep, cattle, and dogs [94,95,96,97]. Specifically, *Salmonella abortus equine (S. abortus equi)* induces equine abortion in pregnant mares during late pregnancy [20]. It also leads to neonatal septicemia and polyarthritis in horses in various countries, including Italy, Great Britain, Argentina, and the United States [98,99]. In recent years, there have been cases of abortion outbreaks among female donkeys in China’s large-scale donkey farms, caused by S. abortus equi. [20,21]. *S. abortus equi* is primarily transmitted through the fecal–oral route and direct contact with infected animals. Infected horses can shed the bacteria in their feces, contaminating the environment and leading to potential ingestion of the pathogen by other susceptible horses. Additionally, direct contact with infected animals, their secretions, or contaminated surfaces can also contribute to the spread of *S. abortus equi.* Control measures for *Salmonella* infection include the use of antibiotics, probiotics, yeasts, and bacteriophages [93,100].

#### 2.2.2. *Streptococcus zooepidemicus*

*Streptococcus equi* subsp. *zooepidemicus (S. zooepidemicus)* causes abortion and respiratory disease in horses and donkeys [101,102]. It can infect a wide range of animals, including humans, monkeys, sheep, dogs, cattle, and swine [103,104], and is associated with various diseases in different species [105]. *S. zooepidemicus*-induced equine ascending placentitis is a common cause of abortion in mares [106]. Recently, Stout et al. found *S. zooepidemicus* to be present in healthy horses with a 55% positive rate using nanoscale real-time PCR detection [107]. Transmission of *S. zooepidemicus* occurs through animal contact or contamination of food or utensils [108]. PCR and ELISA detection are commonly used to diagnose *S. zooepidemicus* infection [109]. 

#### 2.2.3. *Escherichia coli*

*Escherichia coli (E. coli)* is considered a normal part of the gastrointestinal tract in horses. However, many strains of *E. coli* can cause diseases in the gastrointestinal tract, as well as other infections [110]. For example, both *E. coli* and *S. zooepidemicus* are primary pathogens of bacterial placentitis in mares [111]. *E. coli* is an opportunistic pathogen that can cause abortion in donkeys or horses with immune deficiency. Phenotypic identification and PCR detection are commonly used for diagnosing *E. coli* [112,113].

#### 2.2.4. *Rhodococcus equi*

*Rhodococcus equi (R. equi)* causes severe pneumonia in foals and abortion in mares, as it is a member of the actinomycetes [114,115]. Nakamura et al. isolated *R. equi* from an aborted fetus at 196 days of gestation in mares [33]. Additionally, Szeredi et al. described two cases of equine abortion induced by *R. equi* infection at 7–8 months of gestation [34]. Evidence suggests that airborne and foodborne transmission are the primary routes of *R. equi* transmission [116,117]. PCR is used for early detection of *R. equi* infection [118]. Moreover, ELISA can also be used to diagnose *R. equi* infection [119]. 

#### 2.2.5. *Leptospirosis*

Leptospirosis is a significant zoonotic disease that is widely distributed globally. It belongs to the family *Leptospiraceae*, order *Spirochaetales*, genus *Leptospira*, and can infect a wide range of hosts [120,121]. It is closely associated with syndromes and equine reproductive diseases [122]. Consistently, Leptospirosis-induced equine abortions have been reported in numerous cases [123,124]. One study found that donkeys are more susceptible than horses and mules [125]. The primary transmission route of leptospirosis depends on direct contact with infected animals or indirect contact with contaminated urine or water [126]. PCR and the Microscopic Agglutination Test (MAT) are currently convenient methods for diagnosing leptospirosis [127,128]. Thus, early diagnosis and strict implementation of biosecurity measures to limit the spread of these infectious agents within the herd might be helpful in effectively preventing and controlling bacterial abortions in equines.

### 2.3. Fungal Placentitis

Infectious causes are considered the critical factors associated with abortion in equine [25,129]. Consistently, fungal placentitis is caused by *Aspergillus* spp. and *Mucocutaneous* fungi, which also induce abortions in pregnant mares [36,37]. *Aspergillus* spp. is widespread in the environment, such as in animal excreta, soil, and water [130,131]. It is an opportunistic fungal infection in equines with low immunity. PCR and histopathology detection are effective methods for diagnosing fungal placentitis. Additionally, itraconazole has been found to be an effective drug for controlling fungal placentitis [36].

### 2.4. Parasitic Diseases

Neosporosis is closely related to *Toxoplasma gondii* and *Sarcocystis* spp. and belongs to the Apicomplexa phylum of the *Sarcocystidae* family [38]. It can infect various animals, including cattle, camels, ruminants, sheep, dogs, chickens, and horses [132,133,134,135,136,137]. Neosporosis can cause significant economic losses due to reproductive failure [138,139]. Consistently, one study reported a high exposure to *Neospora* spp. in horses and donkeys worldwide [140]. Similarly, another study reported that *Neospora caninum* is a major cause of abortion in donkeys in Iran, with a molecular prevalence of 34.5% in blood samples [141]. The data above demonstrate that Neosporosis has a significant impact on the economic and animal health of the horse and donkey industry. Transplacental transmission plays a crucial role in the epidemiology and circulation of Neosporosis, and the diagnosis is primarily conducted through PCR and serologic tests using enzyme-linked immunosorbent assay. Currently, no effective vaccines for Neosporosis control have been developed, making biosecurity and scientific management the best choices for prevention [133].

Equine piroplasmosis (EP), caused by *Theileria equi* and *Babesia caballi*, affects equids such as horses, donkeys, mules, and zebras. It is a global tick-borne disease that has a significant impact on the horse and donkey industry [142,143]. Horses are more susceptible to EP than donkeys and require greater attention [144]. Some studies have reported cases of abortion in mares caused by *T. equi* in Brazil [145,146]. Additionally, one study found a high positive rate of *T. equi* in domestic and wild donkeys in Israel [147]. The possible transmission routes of EP include transplacental, direct-contact, and tick-borne transmission [148,149]. The diagnosis of EP primarily relies on DNA-based molecular diagnostic techniques such as PCR, microscopy observation, and serological tests using ELISA [150]. Control of the vectors has been the primary strategy for preventing EP [144,151].

## 3. Non-Infectious Risk Factors Associated with Pregnancy Loss 

While infectious agents, such as viruses and bacteria, are the primary causes of equine abortions, there are also several non-infectious risk factors that can contribute to pregnancy loss in equines. These non-infectious risk factors include:

### 3.1. Twinning

Twinning is a major cause of pregnancy loss and abortion in the equine industry, including donkeys. Multiple ovulations contribute to a higher twinning percentage [152], resulting in pregnancy loss during the late months of gestation, when the lack of uterine space and nutrition becomes more obvious [153]. In the past, various measures were taken to reduce twinning in equines, such as eliminating older mares [154,155]. The introduction of ultrasonography and early identification of twins during gestation have been instrumental in reducing abortion in horses [156,157,158]. However, while twinning is a significant cause of abortion in equines, a more effective study of the breeding characteristics of individual jennies and mares is necessary. Consequently, Allen et al. reported that maiden and barren mares also have an increased incidence of twin foals [159]. Mares with foals at foot have a rare chance of having twins due to their high nutrient requirements, and mares with better nutrition have a greater likelihood of having twins. The twinning percentages during foal heat and three consecutive estruses were 15.0, 49.0, 23.0, and 15.0, respectively. The literature indicates that age is not a determining factor for twinning in mares, and abortion can occur at any stage of pregnancy, regardless of age. However, jennies and mares with a history of twinning should be closely monitored during subsequent pregnancies. The main factors contributing to twinning are multiple ovulations and the equine’s nutritional status. 

### 3.2. Early Pregnancy Loss in Aging Equines

Pregnancy loss is a significant factor in equine reproductive medicine and has important implications for animal welfare and the economy. In mares, most pregnancy failures occur between the initial diagnosis and day 65 of gestation, a period commonly referred to as “early pregnancy loss.” In mares, the fertilization rate is not affected by age, but pregnancy loss is significantly higher in aging mares than in young ones [160,161]. Embryonic losses in older mares are much higher compared to young mares, specifically during the 0–6 oviductal days and 6–16 early uterine days [162,163]. Abnormal development of embryos is common in aging mares due to less viable or favorable environments in the oviduct [161].

### 3.3. Progesterone Deficiency

Pregnancy maintenance relies heavily on the secretion of progesterone produced by the corpus luteum (CL), and is essential until the endometrial cup forms around 35 days after ovulation. The developing embryo should migrate through the uterine lumen during the 6–16 days post-ovulation to ensure luteal maintenance and suppress endometrial PGF2α production [164,165], resulting in a condition known as “maternal recognition of pregnancy.” Multiple endometrial cysts may obstruct fetal movement and disrupt corpus luteum maintenance. A potential reason for deficient plasma progesterone concentrations in pregnant mares is the release of a stimulus that triggers PGF2α [164,165].

**Figure 1:**
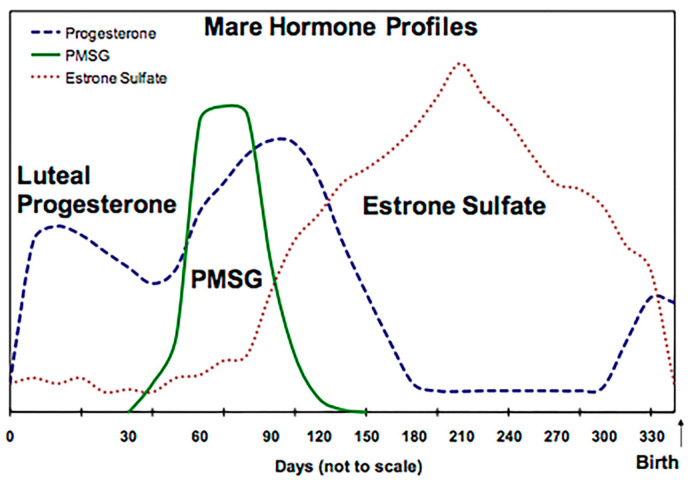
Hormonal profile of pregnant mare [https://www.vet.cornell.edu/animal-health-diagnostic-center/testing/testing-protocols-interpretations/equine-female-reproductive-testing].

Progesterone analysis and pregnancy monitoring can help distinguish various stages of reproduction in mares. As shown in Figure 1, luteal progesterone levels are initially high during the gestation period, reaching their peak concentration at around 120 days of pregnancy and then declining. The corpus luteum produces progesterone to maintain pregnancy up to the fourth or fifth month, after which the placenta secretes sufficient progesterone. 

Tests such as progesterone (P4), pregnant mare serum gonadotropin (PMSG), and estrone sulfate (EIS) can be used individually or in combination to assess pregnancy in horses. These tests are suitable for evaluating conception rates in horses with unidentified pregnancies, such as those that have been rescued, pasture-bred, or bought at auction. PMSG or estrone sulfate can be helpful in determining whether the placenta has been established in early-pregnancy mares. Hormonal treatment can prevent abortion due to circulating progesterone deficiency in horses. External progesterone immunization is one effective method to address low or inadequate maternal serum progesterone levels [166].

### 3.4. Umbilical Cord Torsion

Umbilical cord torsion is a pathological condition characterized by excessive twisting of the cord, leading to partial or complete obstruction of umbilical vessels or the urachus [167]. In this condition, the single vein that enters the fetal abdomen, an amniotic portion of the cord, merges with two umbilical veins that carry oxygenated blood from the placenta [168]. If the umbilical cord wraps around a part of the fetus, suffocation can occur. Jennies and other equine animals should be regularly checked during the last trimester of pregnancy because abnormal tension in fetal reflexes can cause misalignment of the head, neck, and forelimbs, potentially leading to abortion or dystocia. In such cases, the dead fetus is not immediately expelled, and there may be some tissue autolysis [169]. In addition, some stallions produce long cords, and these are more likely to become twisted. Various factors can influence umbilical cord length [170], and the number of twists is unknown [169]. Abortion in mares due to umbilical torsion is a sporadic condition, and fetal complications that lead to abortion are typically a future risk [169,171]. Normal mobility of the equine fetus’s umbilical cord can be observed using ultrasonography [172]. 

### 3.5. Mare Reproductive Loss Syndrome 

Mare reproductive loss syndrome (MRLS), an abortigenic disease, primarily causes early and late fetal losses and is associated with an environmental factor rather than an abortigenic agent [173,174]. MRLS has been reported in several states, including the USA and Australia, and has resulted in significant economic losses in the equine industry [175,176]. For example, McDowell et al. reported that MRLS significantly affected the Ohio River Valley of the United States during the spring of 2001 and 2002, mainly due to Eastern tent caterpillars (ETC) [177,178]. Consistently, Burns et al. found that MRLS was associated with higher temperatures and low humidity [176]. The most effective method of controlling MRLS is to prevent horses, particularly pregnant mares, from being exposed to ETC and possibly other hairy caterpillars [177].

### 3.6. Exposure to Mycotoxins

Mycotoxins, such as aflatoxin, ochratoxin A, fumonisin, zearalenone, and deoxynivalenol, are frequently found as contaminants in animal feedstuff [179]. These mycotoxins can cause reproductive and immune-toxic diseases in various farm animals, including pigs, cows, sheep, horses, and poultry [180,181,182,183]. The diagnosis of mycotoxins primarily relies on PCR, ELISA, flow injection immunoassay (FIIA), chemiluminescence immunoassay (CL assay), lateral flow immunoassay, and flow-through immunoassay [184,185]. Recently, microbial detoxification technology has been widely used for mycotoxin degradation [186].

### 3.7. Managemental Issues

In recent years, extensive feeding management practices, including mechanical injuries and nutritional deficiencies, have been identified as significant causes of abortion in equines [4,7]. Furthermore, road transport in pregnant mares may lead to elevated cortisol due to stress, which may lead to abortion [155]. Thus, it is crucial for veterinarians and practitioners to be aware of these risk factors in order to effectively mitigate their impact (Table 1). By understanding the role of proper management, including feeding and prevention of mechanical injuries, strategies can be developed to prevent abortions and improve overall reproductive performance and productivity in the equine industry. Addressing these non-infectious risk factors through proper management, nutrition, and environmental control can help to reduce the incidence of equine abortions. Regular veterinary check-ups, close monitoring of the mare and donkey’s health, and prompt intervention in case of any complications are crucial in mitigating the impact of these non-infectious factors on equine reproductive success.

## 4. Conclusions

In summary, pregnancy loss has significant economic implications for the equine industry, and can also adversely affect reproductive performance and efficiency in horses and donkeys. This review article examines potential risk factors for pregnancy loss in equines and provides detailed guidance on prevention, diagnosis, treatment, and management. By implementing appropriate management practices and promptly identifying the cause of pregnancy loss, equine reproductive success can be enhanced.

## Figures and Tables

**Table 1:** Summary of infectious and non-infectious causes of equine abortion.

Classification	Causative Agent of Abortion	Clinical Outcomes	Species	References
**Bacterial**	*Salmonella abortus equi*	Abortion	Donkey	[20,22,100]
	*Salmonella abortus equi*	Abortion	Horses	[18]
	*Chlamydia psittaci*	Abortion	Horses	[30,187,188]
	*Neospora*	Abortion	Horses	[189]
	*Escherichia coli*	Abortion	Horses	[190]
	*Mycobacterium avium* subsp.	Abortion	Horses	[111]
	*Streptococcus equi*	Abortion	Horses	[168]
**Viral**	EHV-4	Abortion, neurological and respiratory diseases	Donkey	[191]
	EHV-7	Abortion in the first to second trimester	Donkey	[70]
	EHV-8	Abortion, anorexia, sadness, unwillingness to move	Donkey	[71]
	Equus caballus papillomavirus 2(EcPV)	Abortion	Horses	[192]
	EHV-1	Abortion, neonatal deaths	Horses	[1,48,49,193,194]
	EHV-2	Abortion, dyspnea, respiratory and neurological symptoms	Horses	[195,196]
	EHV-4	Abortion	Horses	[197]
	EHV-5	Abortion	Horses	[198]
	EAV	Abortion	Horses	[199]
**Fungal**	*Leptospira*	Abortion, stillbirth	Horses	[200,201]
	*Aspergillus*	Abortion, classic signs of placentitis (premature udder development and milk dripping), stillbirth	Horses	[2,36,124]
	*Chlamydia*	Abortion	Horses	[202]
	*Chlamydia psittaci*	Abortion	Horses	[203]
**Parasitic**	*Toxoplasma gondii*	Abortion	Donkey	[204]
	*Trypanosoma equiperdum*	Abortion, emaciation	Horses	[205]
	*Neospora caninum*	Aborted fetus	Horses	[206]
**Management**	Feeding mismanagement	MRLS-type abortions	Horses	[177]
	Transport of late-pregnant mares	Induced cortisol and abortion	Horses	[155]
**Physiological changes**	Torsion of umbilical cord	Abortion, stillbirth	Horses	[160,207]
	Excessive length or shortness of the umbilical cord	Abortion	Horses	[208]
	Twin pregnancy	Abortion followed by uterine prolapse	Horses	[152]

## Data Availability

All the data are available in the manuscript.
